# The relationship between protective factors and common mental disorders among female urban slum dwellers in Ibadan, Nigeria

**DOI:** 10.1371/journal.pone.0263703

**Published:** 2022-02-08

**Authors:** Olutoyin Sekoni, Sumaya Mall, Nicola Christofides

**Affiliations:** 1 Department of Community Medicine, College of Medicine, University of Ibadan, Ibadan, Oyo State, Nigeria; 2 School of Public Health, University of the Witwatersrand, Johannesburg, Guateng, South Africa; Gachon University Gil Medical Center, REPUBLIC OF KOREA

## Abstract

**Background:**

On the African continent, many people live in conditions of adversity known to be associated with the onset of mental disorders, yet not all develop a mental disorder. The prevalence of common mental disorders such as depression and anxiety in the general population of Nigeria is comparatively low. Prevalence data of mental disorders in slum settings in Nigeria is sparse. There is a need to better understand the relationship between protective factors and the occurrence of common mental disorders in the Nigerian slum context. This study aimed to describe the relationship between protective factors and the occurrence of common mental disorders among female urban slum dwellers in Ibadan, Nigeria.

**Methods and findings:**

A cross sectional household survey of 550 women was conducted in slum settlements in Ibadan, Nigeria. Interviewer administered questionnaires were completed to elicit information on protective factors (social connectedness, self-esteem, social support, resilience) and common mental disorders (depression, anxiety and stress). The DASS-21 was used to measure common mental disorders and protective factors were measured using the Social Connectedness Scale, Multidimensional Scale of Perceived Social Support, Resilience scale and the Rosenberg Self Esteem Scale. A multivariable logistic regression model was employed to examine associations while adjusting for relevant confounders. Common mental disorders were reported by 14.0% of the respondents. Resilience and social support were found to be protective against reporting symptoms of common mental disorders. Women who reported higher levels of social support and resilience were less likely to report common mental disorders (OR:0.96, 95% CI 0.93, 0.98) and (OR:0.95, 95% CI 0.91, 0.99) respectively. Women who were 65 years and older were also less likely to report the occurrence of common mental disorders (OR:0.38, 95% CI 0.15, 0.98) compared to those aged 18–34 years.

**Conclusion:**

Social support and resilience appear to be protective against common mental disorders among these respondents. Further research should be conducted to explore the pathways through which protective factors reduce the likelihood of the occurrence of common mental disorders. This would be important in the development of mental health interventions.

## Introduction

In Low and Middle Income Countries (LMIC) including in sub-Saharan Africa, mental disorders are common and may be exacerbated by overburdened or fragmented health systems, high patient load and few psychiatrists, psychologists and other mental health professionals [1]. In addition, virtually all LMIC have significant slum populations where there are higher levels of mental illness, and poorer outcomes [2]. Research from slums in India and Bangladesh suggests high prevalence of mental disorders with 75% of adolescents in the slums of India reporting at least one type of mental disorder, which was similar to what was found among slum dwelling adults in Bangladesh [3, 4]. With the estimated increase in the population of slums across the world in the coming years, there is a corresponding potential for an increase in the number of individuals at risk of common mental disorders within slum populations [5].

Women are at greater risk of developing common mental disorders, a finding which has been consistently highlighted in the literature [6–8]. The cause for the gender variation is not entirely clear but evidence has pointed towards the role that hormones may play as well as a greater prevalence of internalizing symptoms among women which serve as triggers for mental disorders [9]. Reproductive hormones and stress hormones fluctuate in women over the life course starting from puberty, during pregnancy and the postpartum period as well as the perimenopausal period and can predispose to the development of common mental disorders in vulnerable women [10].

The prevalence of common mental disorders among Nigerian women has been examined through various lenses. The prevalence of depression has been found to range from 27.8% to 42.5% among women that are HIV positive, postpartum women, victims of IPV and among infertile women [11–15]. Similarly, the prevalence of anxiety among Nigerian women has ranged between 5.6% and 33.3% among female victims of intimate partner violence, women within the community and among women postpartum [12–14, 16]. Evidence in the literature with respect to stress among Nigerian women is limited as existing research has focused mainly on occupational settings [17–19]. However, Adamu et al reported moderate levels of perceived stress among HIV positive women [20].

Adversity and poverty, which are characteristics of slum environments, have been associated with the onset of mental disorders such as depression, anxiety and stress [21]. Lower income and poorer housing, lower levels of education and food insecurity are also associated with mental disorders, although the relationship is not consistently reported [22–26]. There is also a link between women’s socioeconomic status and their mental health with women of lower socioeconomic status being at greater risk of mental disorders than those of higher socioeconomic status [27, 28].

The literature has also suggested that etiopathological factors such as environmental, personal and biochemical factors have a role to play in the onset of common mental disorders. These could range from factors such as maternal physiological stress in pregnancy, nutritional deficiency and environmental pollution to the state of biochemical factors within the body such as cortisol levels [29–32].

Over the past decade, in addition to research to understand risks and extent of mental disorders, there have been studies exploring protective factors which may reduce the risk of developing mental disorders, including social support, social connectedness and resilience [33–38]. Resilience has been described as a function of active coping which in turn makes it possible for an individual to navigate conditions of adversity [39, 40]. A plethora of research has examined factors such as social support and social connectedness and how these might be protective against the occurrence of common mental disorders in various settings [35, 36, 38, 41, 42]. Systematic reviews on the relationship between social support and social relationships on mental health suggest that social support across the life course may protect against depression with a strong inverse relationship [42–44]. Social connectedness and resilience have also been found to be protective against common mental disorders [45, 46].

Various factors have been associated with an individual’s ability to cope and be resilient in the adverse conditions of the slum environment. For example, in research among adolescents in the slums of Kenya and among children in South Africa and Malawi, resilience was associated with social support and a nurturing environment of care and encouragement as well as positive individual attributes such as social competence, critical thinking and autonomy [47, 48]. However, researchers also point out that multiple factors interacting at various ecological levels are responsible for making an individual resilient [49, 50]. Other researchers have also found social support and resilience to be predictive of mental well-being [51, 52].

The prevalence of common mental disorders in Nigeria has varied widely. While depression in community-based surveys in Nigeria has been found to be quite low (5.5%), [53], there are variations for sub-populations. For instance, among individuals with varying clinical condition’s such as stroke, hypertension, epilepsy and lymphatic filariasis the prevalence of depression ranged between 20.0–42.9% [54–57]. Similar findings have been observed in the prevalence of anxiety within the general population compared to specific subgroups in Nigeria. A community-based prevalence of anxiety was reported to be between 3.5% and 4.1% [53, 58]. However, in sub-groups such as medical students and adolescents the prevalence of anxiety was found to be 28.8% and 34.1% respectively [59–61]. A systematic review of stress among health workers in Nigeria reported a prevalence of 61.9% and among undergraduate medical students stress levels were similarly high (59.8%) [19, 62].

There are limited studies in Nigeria that have explored the relationship between protective factors and common mental disorders. Among medical students in Nigeria, an inverse relationship was found between the occurrence of common mental disorders and religiosity and social support. Similar findings were reported among elderly Nigerians [60, 63].

Research into the health of slum inhabitants has become increasingly important considering the fact that almost two billion people would be living in slums globally by the year 2030 [64, 65]. In addition, the sustainable development goals three, six, nine and eleven speak both directly and indirectly to the need to promote the health and well-being of slum inhabitants [66, 67]. In Nigeria, all large cities are characterized by the existence of slums which arise majorly from two types of slum formation. Either as traditional slums located in the city centre or the peri-urban slums located at the outer core of the city. Currently, about 57.7 million Nigerians live in slums [68]. The number of people living in slums is expected to increase over the next decade [68, 69].

Mental health research among slum communities in Nigeria is particularly sparse. One study found that the prevalence of depression among adolescents in an urban slum in South West Nigeria was 29.4% and that social support was associated with better mental health outcomes [70]. Understanding the experience of common mental disorders in slum environments and the role that protective factors may play are important in this context. Our study was conducted to extend the literature on common mental disorders in slum environments and to explore the relationship between common mental disorders and protective factors among female urban slum dwellers in Ibadan, Nigeria.

## Methods

### Study design and setting

We conducted a community based cross-sectional study in slum areas of Ibadan in Oyo State, Southwest Nigeria in October 2018. Ibadan, the capital of Oyo State, is one of the largest cities in West Africa with 13 identified slum areas based on the UN Habitat classification of slums [71]. Five out of the thirteen identified slums were selected randomly for the study, as it was not feasible to include all and the characteristics of the slums are similar. The slums are located within the central core area of Ibadan metropolis and are characterized by poor housing conditions and a haphazard cluster of houses made of mud, plastered with cement, with rusted corrugated iron roofs. Municipal facilities such as running water, waste disposal and sanitation services are non-existent and there are no access roads between dwellings [69, 72, 73]. Levels of literacy are low and most of the residents have low socioeconomic status [69, 72, 73]. Houses are commonly comprised of multigenerational households and represent a homogenous community of individuals [73].

### Study population

The study population consisted of adult women above the age of 18 years who were resident members of the selected households during the period October to November 2018. For a respondent to be eligible to participate in the study she was 18 years or older and a regular resident of the household. Regular residence was defined in line with similar research as spending at least four nights a week in the household for at least one year [74]. A minimum sample size of 418 women was determined based on sample size calculations utilising a prevalence of depression of 5.5% among a community sample in Nigeria [53] and adjusting for a 10% non-response. However, as the data for this study was part of larger study that examined other variables and required a larger sample size to achieve other stated objectives with adequate power, a final sample size of 550 was utilised.

### Sampling

A multistage sampling technique was employed. A simple random sampling of five of thirteen slum settlements in Ibadan was generated through the use of a table of random numbers. For the purposes of this study, Enumeration Areas (EAs) which are clearly delineated areas commonly utilised in the conduct of censuses in Nigeria were used as primary sampling units. The EAs are demarcated by the National Population Commission [75]. From the five selected slum settlements, one third of the EAs were selected randomly giving a total of 42 EAs. However this number was increased to 68 in order to ensure that there were enough households available per EA to select just one eligible respondent per household while still bearing in mind the required sample size. Weights used in the analysis were generated and applied based on population parameters for the slum settlements and the enumeration areas.

The NDHS 2013 report which is a nationally representative survey indicated 48 households per EA (54). However, we assumed an average of 60 households per EA (four households per house in 15 houses per EA) given that slum settings are characterized by higher population density. One household was selected randomly by balloting per house where there were at least two households while one adult woman was selected randomly by balloting.

### Procedures

Six female research assistants who had attained tertiary level education collected data through face-to-face interviews using standardized questionnaires programmed using Open Data Kit software uploaded on mobile devices. Training of research assistants took place over five days and they were trained in research ethics, the standard operating procedures for recruitment and conducting interviews and well as the key topics relevant for the study. A distress protocol was implemented and research assistants were trained to identify participants in distress and to provide referrals for immediate care to a clinical psychologist or psychiatrist should this be required. A small sachet of detergent was given to each participant as a token of appreciation for participation in the study.

In addition to the administration of questionnaires, research assistants were also trained to review questionnaires for completeness as well as maintenance of ethical standards. Training on the Open Data Kit was also carried out to teach the research assistants how to use the software and upload the data once obtained.

The questionnaire was translated to the most-commonly spoken language in the slums, Yoruba, and back translated to ensure that original meanings were preserved [76]. The questionnaire was pre-tested in an enumeration area within a slum area that had not been sampled. All detected ambiguities were removed and corrections made following analysis of the pre-test.

### Ethical approval

Ethical approval for the study was obtained from the University Of Witwatersrand Faculty of Health Science Human Ethics Committee as well as the Ethics Review Board of the Oyo State Ministry of Health, Oyo State, Nigeria. Permission to conduct the study was also obtained from appropriate local community authorities. Respondents were given information on the study and participation was voluntary. Written informed consent was obtained from all respondents. Interviews were conducted in private to ensure confidentiality.

### Measures

Items of interest explored included common mental disorders with specific reference to depression, anxiety and stress, the presence of protective factors (social connectedness, self—esteem, perceived social support and resilience) as well as sociodemographic characteristics.

### Outcome variable

The outcome variable for this study was the occurrence of common mental disorders. This was assessed using the Depression Anxiety and Stress Scale (DASS-21), a short version of the original 42 item depression, anxiety and stress scale. The internal consistency was found to be acceptable with a Cronbach’s alpha of 0.73. Based on an exploratory factor analysis we found that the individual sub-scales for depression, anxiety and stress did not load on the three subscales as intended by the developers of the instrument [77]. A previous study in Nigeria had reported high validity and reliability of the DASS in Nigeria among medical students [78]. This study reported good internal consistency for both the overall instrument as well as the subscales. Since this was not the case in our study, we decided not to use the subscales but instrument as a whole which measured the symptoms of common mental disorders.

### Protective factors

Social connectedness was measured using the Social Connectedness Scale (Revised). This is a revision of the original 8 item scale to a more robust and encompassing version [79]. The revised version is a 20 item scale rated on a 6 point Likert type scale ranging from 1 = strongly disagree to 6 = strongly agree with ten positive and ten negative questions and a total score range of 20 to 120 [80]. Respondents were asked statements that reflect various ways in which they had viewed themselves within the preceding week. Higher scores indicate higher levels of social connectedness. Our examination of the internal consistency of this scale revealed it to be good with a Cronbach’s Alpha of 0.81.

Self- esteem was measured using the Rosenberg self-esteem scale. It is a 10 item scale that identifies global self-worth by measuring both positive and negative feelings about the self with responses provided on a 4 point Likert scale of ‘strongly agree’ to ‘strongly disagree [81]. Higher scores indicate higher levels of self-esteem. The scale has been favoured for use by several researchers and is noted to have undergone several rounds of psychometric analysis and validation lending further credence to its utility [82–84]. Several researchers also tend to favour treating self-esteem as a continuous variable from the scores obtained from the self -esteem scale which is what we also adopted [85–87]. In this study, we found the internal consistency to be fair with a Cronbach’s Alpha of 0.73.

For social support we used the Multidimensional Scale of Perceived Social Support developed by Zimet and colleagues in 1988 [88]. This is a 12-item instrument that measures perceptions of support from family, friends and a significant other on a 7 point Likert scale. Total scores range from 12 to 84. Higher scores indicate higher levels of perceived social support. It has also been noted to have good reliability and validity and has been used by other researchers [89–92]. In order to enhance precision, we treated social support as a continuous rather than a categorical variable. We found good internal consistency with a Cronbach’s alpha of 0.89.

Resilience was measured using the resilience scale developed by Wagnild and Young and which is a summated reporting scale that utilizes a 7 point Likert format [93]. It has been previously used in Nigeria [94]. The total resilience score ranges between 25 and 175 points an in our study, the resilience scale had good internal consistency with a Cronbach’s alpha of 0.88.

### Sociodemographic characteristics

Sociodemographic characteristics: these included age, educational status, marital status, employment status, occupation and wealth index. Wealth index was assessed using a measure of relative wealth for urban populations derived from nationally representative survey data [95].

### Statistical analysis

Data were analyzed using STATA version 14 [96]. The analysis was cross sectional and all independent variables were between subjects. No participants were missing data for either DASS-21or the protective factors. We confirmed the internal consistency of scales using a Cronbach’s alpha. We adjusted for the cluster sampling during analysis using the *svyset* command in STATA. We also weighted the data to ensure that it was representative of the Ibadan slums using *pweight*. Weights used in the analysis were generated and applied based on population parameters for the slum settlements and the enumeration areas. The weights were calculated by taking the inverse of the probability of selection for each individual in the data sample. We generated a dichotomous variable, with those who had any symptoms of common mental disorders as “1” and those that did not have any symptoms of common mental disorders as “0”. We calculated medians and the interquartile range for all protective factors. We calculated the odds ratios and 95% confidence intervals for all protective factors. Multivariable binary logistic regression model was employed to examine the relationship between protective factors (social support, resilience, self-esteem and social connectedness) and the occurrence of common mental disorders while controlling for relevant socio-demographic factors such as age and marital status. We assessed the goodness of fit using the Hosmer and Lemeshow’s test and found that our model fitted the data well. All analyses were conducted at 5% level of significance.

## Results

The median age of women in our study was 40 years with an interquartile range of 30–55 years. About a fifth had not acquired any formal schooling (23.3%) and almost two thirds were married/cohabiting (65.5%). The majority had been employed during the preceding three months (76.0%) and reported low socio-economic status with 72.8% categorized as being from the poor and poorest urban wealth quintiles (Table 1).

**Table 1:** Sociodemographic characteristics.

Characteristic	Frequency	Unweighted percentage (%)	Weighted percentage (%)
**Age at last birthday (n = 550)**			
18–34	198	36.00	37.21
35–49	169	30.73	29.99
50–64	92	16.73	16.98
≥65	91	16.55	15.83
**Median age(median, IQR** ^a^ **)**	**40(IQR:30–55)**		
**Educational status (n = 550)**			
None	123	22.36	23.36
Any primary	165	30.00	28.38
Some secondary	67	12.18	12.58
Completed secondary/tertiary	195	35.45	35.69
**Marital status**			
Married/cohabiting	373	67.82	65.52
Single/never married	43	7.82	9.39
Separated/divorced/widowed	134	24.36	25.09
**Employed in the last 3 months (n = 550)**			
Yes	425	77.27	76.02
No	125	22.73	23.98
**Alcohol use in the past 12 months (n = 550)**			
Yes	35	6.36	5.33
No	515	93.64	94.67
**Urban Wealth index quintiles (based on assessment of household assets) (n = 550)**			
Poorest	184	33.45	33.43
Poor	210	38.18	39.44
Slightly wealthier	156	28.36	27.14

^a^IQR: Inter-quartile range.

The majority of the women did not report any mental health symptoms. Overall, only 14.0% reported any symptom of a common mental disorder (Table 2).

**Table 2:** Mental health status.

Mental health	Frequency	Weighted percentage (%)
**Mental health status**		
Any mental health symptoms	77	14.00
No mental health symptoms	473	86.00

Majority of the respondents reported protective factors. The median social connectedness score was 97 (IQR: 88–101) while for self esteem the median was 30 (IQR: 29–32). Social support and resilience had median scores of 62(IQR: 53–72) and 142 (IQR:137–148) respectively (Table 3).

**Table 3:** Scores for protective factors: Social connectedness, self esteem, perceived social support and resilience.

Protective factors	Median	Interquartile Range
**Social connectedness**		
Social connectedness score	97	88–101
**Self Esteem**		
Self Esteem score	30	29–32
**Social Support**		
Social Support score	62	53–72
**Resilience**		
Resilience score	142	137–148

Unadjusted odds ratios of age group, marital status, education, wealth index as well as the protective factors of social support, resilience, self esteem and social connectedness with common mental disorders are presented together with the fully adjusted model in Table 4. No associations were found between age group, marital status, education, wealth index, self esteem, resilience, social connectedness and common mental disorders.

**Table 4:** Unadjusted odds ratios and fully adjusted multiple regression model of protective factors and common mental disorders (N = 495).

Variable	OR(95% CI)	AOR (95%CI)
Social Support	0.96 (0.93–0.98)*	0.96 (0.93–0.98)*
Resilience	0.96 (0.92–1.01)	0.95 (0.91–0.99)*
Self Esteem	1.04 (0.96–1.12)	1.09 (0.99–1.19)
Social Connectedness	0.99 (0.94–1.04)	-
**Age group**:		
18–34	Ref.	
35–49	0.91(0.47–1.75)	0.75(0.41–1.37)
50–64	1.28(0.56–2.89)	1.01(0.40–2.55)
≥65	0.88(0.48–1.63)	0.38(0.15–0.98)*
**Marital status**:		
Married/cohabiting	Ref.	
Single/never married	1.43(0.63–3.23)	1.38(0.51–3.72)
Separated/divorced/widowed	1.35(0.73–2.49)	1.33(0.56–3.15)
**Education**:		
None	Ref.	
Any primary	0.69(0.40–1.17)	-
Some secondary	0.87(0.45–1.67)	
Completed secondary/tertiary	0.65(0.39–1.09)	
**Urban Wealth index**:		
Poorest	Ref.	
Poor	0.78(0.44–1.38)	-
Slightly wealthier	0.73(0.35–1.52)	

p≤ 0.05**.

In the adjusted model, higher social support, higher levels of resilience and older age were all found to be protective against the occurrence of common mental disorders. For every one point higher on the social support scale, women were 4% less likely to report a mental disorder. Similarly, for every point higher on the resilience scale women were 5% less likely to report a mental disorder. Women aged 65 or older were 12% less likely to report any common mental disorder than women aged 18 to 34 years of age. There were no differences in self-esteem among those reporting symptoms of mental disorders and women who did not report any mental health symptoms.

## Discussion

Our study set out to examine the prevalence of common mental disorders and the relationship between protective factors and reporting symptoms of anxiety, depression and stress among female urban slum dwellers in Ibadan, Nigeria. This was achieved through a cross sectional study design appropriate to estimate prevalence. The study had an adequate sample size with sufficient power and a representative sample. The prevalence of common mental disorders was found to be 14.0%. Protective factors against the occurrence of common mental disorders included resilience, social support and older age. There was no association between self-esteem or social connectedness with the occurrence of any common mental disorder in our study.

In the slums in Ibadan, despite poor living conditions and a context characterized by limited social protection or services, we found low levels of common mental disorders. This finding is not consistent with what has been reported in other places where those living in poor urban neighbourhoods in Bangladesh and Ghana were found to have high levels of common mental disorders [3, 97]. One of the possible explanations for the low levels of common mental disorders in our study is the homogeneity and long history of the traditional slums. Many slums elsewhere in sub-Saharan Africa, such as those in Kenya and South Africa, are receiving areas for migrants and are characterized by diversity in ethnicity and culture and as a result, lower levels of trust and cohesion [98]. High levels of social cohesion are present in the inner city Ibadan slums. Social cohesion has been found to be positively associated with mental well-being suggesting that higher homogeneity in communities may be protective against common mental disorders such as depression and anxiety [98–100]. For instance, ethnic density or homogeneity within migrant communities in high income countries has been associated with lower levels of mental disorders [101]. The reason that ethnic density is protective against mental disorders is that it fortifies an individual’s sense of self-identity since this is generally defined by the perceptions of others within their immediate environment. Hence individuals see themselves as being similar to those in their environment which fosters greater self-esteem [102–104].

As reported elsewhere, the women who participated in our study reported very high levels of social support which was associated with lower odds of common mental disorders. Informal neighbourhoods in Nigeria and Bangladesh where social networks and social support have been identified as strong, have been found to moderate the effects of adversity, thereby promoting mental health [105, 106]. Within the Ibadan slums, poverty and limited access to opportunities are a common experience and may represent a rallying point to which women in the communities can identify and accept. This phenomenon has been described as fatalism [107]. While social support was protective against common mental disorders we did not find any association between social connectedness and common mental disorders among our respondents. A reason for this might be that because there were such high levels of social connection among this group, not much variability was observed in the data and hence it was not significantly associated with the occurrence of common mental disorders. Another reason could be that the measure was not validated previously in slum settings. It is also possible that social support might be more important in protecting against common mental disorders than social connectedness which might highlight the value of social quality over quantity.

Resilience was also found to be protective against common mental disorders among our sample of women. This finding corroborates the results from other studies. Coope et al found resilience to be protective of mental health in their review among internal migrants in low and middle income countries and this was also seen by Wu et al in their review among both cross border and internal migrant youth across several countries [52, 108]). Our findings of the protective nature of resilience contribute to the literature on the role that resilience might play in the promotion of mental health. There is evidence to suggest that interventions to improve resilience are associated with lower rates of common mental disorders as reported by Mathias and colleagues in their study among female slum dwellers in India [109].

Researchers from other parts of Africa have reported associations between the occurrence of common mental disorders and lower educational levels, unemployment and poverty [97, 110, 111].

We acknowledge limitations in this study. Recall bias cannot be entirely ruled out as respondents were required to provide information about experiences that occurred in the past. In addition, being a cross-sectional study, the temporal nature of the associations cannot be ascertained. Social desirability bias must also be considered as a limitation as respondents may have under-reported symptoms of mental disorders given the stigma attached to mental illness in the study setting. Finally, we used the DASS as a whole rather than attempting to use the subscales for depression, anxiety and stress as the internal consistency was not optimal.

In spite of acknowledged limitations, the results of this study have implications for the development of mental health interventions in slum settings. Protective factors as observed in this study may represent drivers for the promotion of positive mental health as has been suggested by other researchers [112].

### Conclusion

Low levels of common mental disorders reported among women in the inner-city slum setting suggest that the homogeneity among slum inhabitants may play a role in their mental health. In addition, our study identified associations between protective factors (social support and resilience) and common mental disorders as well age and common mental disorders. Further epidemiological studies are recommended to isolate these factors and their relationship to mental disorders. Programmatic interventions to enhance resilience and social support are also recommended but may not be sufficient. Given the high levels of poverty, lack of services and infrastructure in the slum setting, structural changes to address these issues are essential if the well-being of women in the slums and all Nigerians as a whole is to be assured.

## Abbreviations

AOR: Adjusted Odds Ratio
DASS-21: Depression Anxiety and Stress Scale-21
EAs: Enumeration Areas
OR: Odds Ratio
